# Absence of glaucoma in DBA/2J mice homozygous for wild-type versions of *Gpnmb *and *Tyrp1*

**DOI:** 10.1186/1471-2156-8-45

**Published:** 2007-07-03

**Authors:** Gareth R Howell, Richard T Libby, Jeffrey K Marchant, Lawriston A Wilson, Ioan M Cosma, Richard S Smith, Michael G Anderson, Simon WM John

**Affiliations:** 1 The Jackson Laboratory, 600 Main Street, Bar Harbor, Maine, USA; 2 Department of Anatomy and Cell Biology, Tufts University of Medicine, Boston, MA, USA; 3 The Howard Hughes Medical Institute, Bar Harbor, Maine, USA; 4 Department of Physiology and Biophysics, The University of Iowa, Iowa City, IA, USA; 5 Department of Ophthalmology, Tufts University of Medicine, Boston, MA, USA; 6 University of Rochester Eye Institute, University of Rochester Medical Center, Rochester, NY, USA

## Abstract

**Background:**

The glaucomas are a common but incompletely understood group of diseases. DBA/2J mice develop a pigment liberating iris disease that ultimately causes elevated intraocular pressure (IOP) and glaucoma. We have shown previously that mutations in two genes, *Gpnmb *and *Tyrp1*, initiate the iris disease. However, mechanisms involved in the subsequent IOP elevation and optic nerve degeneration remain unclear.

**Results:**

Here we present new mouse strains with *Gpnmb *and/or *Tyrp1 *genes of normal function and with a DBA/2J genetic background. These strains do not develop elevated IOP or glaucoma with age.

**Conclusion:**

These strains provide much needed controls for studying pathogenic mechanisms of glaucoma using DBA/2J mice. Given the involvement of *Gpnmb *and/or *Tyrp1 *in areas such as immunology and tumor development and progression, these strains are also important in other research fields.

## Background

Glaucomas are heterogeneous diseases that affect 70 million people worldwide [1]. They are characterized by the loss of retinal ganglion cells and degeneration of the optic nerve [2,3]. Glaucoma is often associated with an age-related elevation of intraocular pressure (IOP) [4]. Elevated IOP induces glaucomatous neurodegeneration in susceptible individuals. IOP elevation involves compromised drainage of aqueous humor (AqH) through the ocular drainage structures in the iridocorneal angle (angle) [5]. Nevertheless, the molecular etiology of IOP elevation varies between individuals and with the type of glaucoma. In pigmentary glaucoma, the iris is damaged resulting in dispersal of iris pigment and debris into the anterior chamber and AqH drainage structures. In susceptible individuals, the pigment and/or debris induces a process(es) that damages the drainage structures resulting in high IOP and subsequent glaucoma [6-8]. DBA/2J (D2) mice inherit a disease with similarities to human pigmentary glaucoma [9,10], including ultrastructurally abnormal melanosomes [11]. Around 5–6 months of age, D2 mice develop a pigment dispersing iris disease. Pigment and/or iris debris induces drainage structure damage that disrupts the outflow of aqueous humor. This causes an elevation of IOP that first occurs in a significant portion of mice around 8–9 months of age [10]. In our colony, about 70% of D2 mice develop moderate or severe glaucoma by 12 months of age [10]. There are now a number of independent reports on DBA/2 glaucoma including several recent ones (e.g. [10,12-14]). Some but not all of these reports suggest earlier onset damage than we have reported. This earlier onset damage clearly does not occur in our colony as we have studied large numbers of mice [10]. We have previously discussed possible explanations for these differences including the exact substrain used and environmental differences.

We have previously shown that the D2 iris disease is genetically separable into two distinct traits, iris pigment dispersion (IPD) and iris stromal atrophy (ISA) [15]. IPD is characterized by a breakdown of the posterior iris pigment epithelium, slit-like transillumination and prominent pigment dispersion. IPD is caused by a mutation in the glycoprotein (transmembrane) nmb gene (*Gpnmb*^*R150X*^), a recent mutation that arose on D2 in the early 1980's [10,16]. ISA is characterized by deterioration of the iris stroma, and an accumulation of stromal pigment and cell debris in the drainage structures [15], and is caused by a recessive mutation in the tyrosinase related protein 1 gene (*Tyrp1*^*b*^) [16]. D2 mice are homozygous for both *Gpnmb*^*R150X *^and *Tyrp1*^b ^mutations and inherit a much more severe iris disease and subsequent glaucoma than mice carrying either of the two mutations alone [16]. The published characterization of both IPD and ISA was necessarily performed using mice of a mixed genetic background when mapping and identifying the genes that underlie these traits. These phenotypes have never been fully analyzed in the context of a D2 genetic background.

D2 is one of the most widely used mouse strains in glaucoma research. Here, we describe the development of novel strains that can be used in conjunction with D2 to further our understanding of the complex mechanisms involved in pigment dispersing iris disease, IOP elevation and glaucoma. Prior to developing these strains, there were no control, age and strain-matched DBA/2J mice that did not develop these phenotypes. Control D2.B6-*Tyrp1*^*B6*^*Gpnmb*^*B6*^/Sj mice (hereafter referred to as D2.*Tyrp1*^*B6*^*Gpnmb*^*B6*^) are homozygous for wild type alleles of *Tyrp1 *and *Gpnmb *that are derived from strain C57BL/6J (B6). These mice do not develop any iris disease, IOP elevation or glaucoma. DBA/2J-*Gpnmb*^+^/Sj mice (hereafter referred to as D2-*Gpnmb*^+^) are homozygous for a wild-type allele of *Gpnmb *on a D2 genetic background. This *Gpnmb*^+ ^allele is the original D2 allele that predates the *R150X *mutation (See Methods). These mice develop a very mild form of iris disease due to the *Tyrp1*^*b *^mutation, but do not develop elevated IOP or glaucoma. These strains further define the relative contributions of the *Tyrp1 *and *Gpnmb *genes to the D2 phenotype and will serve as powerful control strains in future studies of glaucoma.

## Results

**Figure 1:**
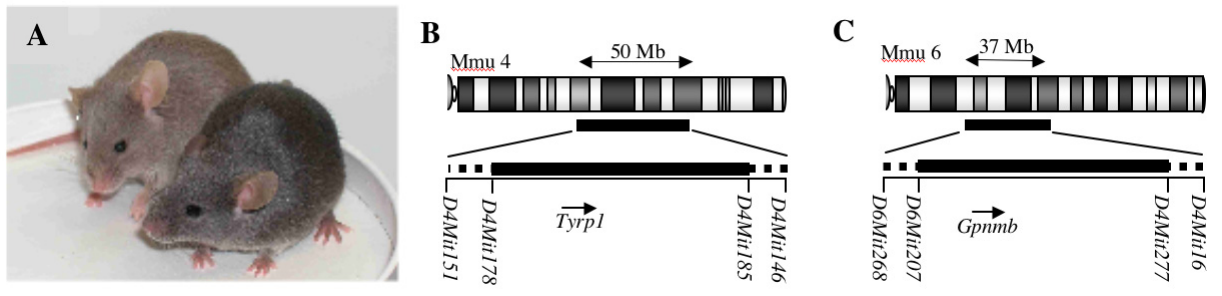
Analysis of D2.*Tyrp1*^*B6*^*Gpnmb*^*B6 *^mice. **A**. D2.*Tyrp1*^*B6*^*Gpnmb*^*B6 *^mice (right) show a darker coat color compared to D2 mice (left) due to the presence of a wild-type *Tyrp1 *gene. **B-C**. Regions of B6-derived sequence containing *Tyrp1 *(**B**) and *Gpnmb *(**C**) are indicated by the black horizontal lines, dotted lines indicate the region within which the breakpoints lie. The estimated maximum sizes of the regions (taken from Ensembl) are indicated. Polymorphic markers flanking the breakpoint regions are shown and the orientation of the genes are indicated below.

We have developed control strains that will maximize the potential of D2 mice in understanding the pathogenesis of both pigment dispersing iris disease and glaucoma. For the first control strain, named D2.*Tyrp1*^*B6*^*Gpnmb*^*B6*^, the mutated forms of *Tyrp1 *and *Gpnmb *were replaced with wild-type alleles from B6 (*Tyrp1*^*B6 *^and *Gpnmb*^*B6*^, see Methods). Mutations in *Tyrp1 *alter coat color in various strains [17]. As a consequence of the *Tyrp1*^*B6 *^allele, D2.*Tyrp1*^*B6*^*Gpnmb*^*B6 *^mice have a darker coat color than D2 mice (Figure 1a). The extent of B6-derived sequence flanking both *Tyrp1 *and *Gpnmb *genes in this strain was assessed using polymorphic markers. For *Tyrp1*^*B6*^, the proximal breakpoint was mapped to between *D4Mit151 *and *D4Mit178 *and the distal breakpoint to between *D4Mit185 *and *D4Mit146 *(Figure 1b). The maximum size of the B6-derived region flanking *Tyrp1*^*B6 *^is 50 Mb or 1.9% of the mouse genome. For *Gpnmb*^*B6*^, the proximal breakpoint was mapped to between *D6Mit268 *and *D6Mit207*, and the distal breakpoint to between *D6Mit277 *and *D6Mit16 *(Figure 1c). The maximum amount of B6 sequence flanking *Gpnmb*^*B6 *^is estimated to be 36 Mb or 1.4% of the mouse genome.

### D2.Tyrp1^*B6*^Gpnmb^*B6 *^show no iris disease, IOP elevation or glaucoma

**Figure 2:**
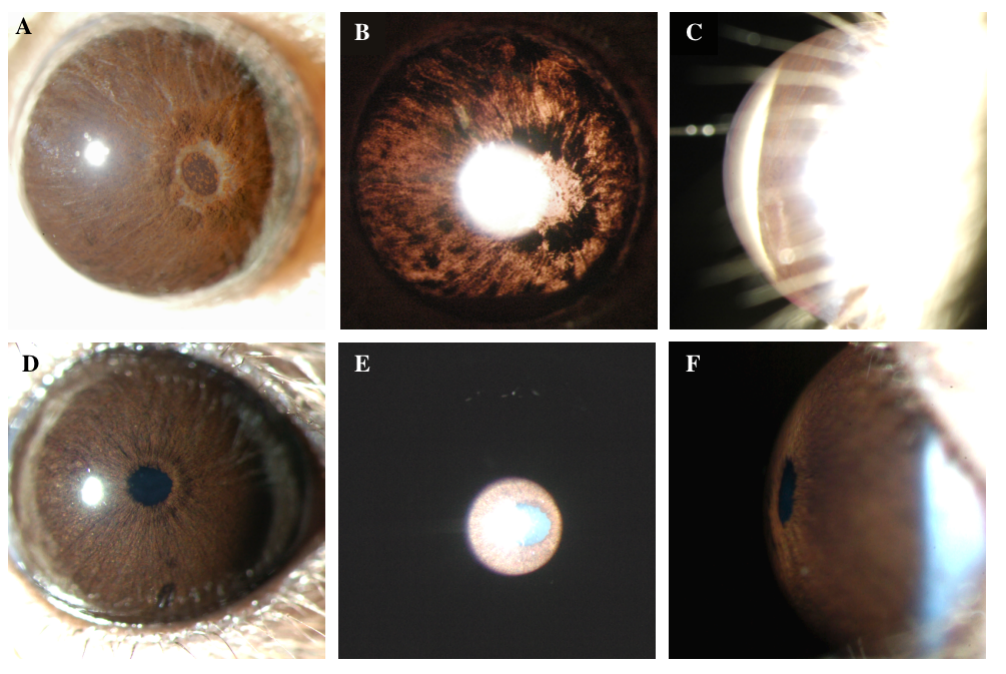
Comparison of iris disease in 13 months old D2 mice and D2.*Tyrp1*^*B6*^*Gpnmb*^*B6 *^mice. **A **and **D **show broad-beam illumination, **B **and **E **show transillumination defects and **C **and **F **show the relative dimensions of the anterior chamber. **A-C**. In D2 mice, the iris disease involves iris stromal atrophy, transillumination defects and progressive depigmentation with abnormal dispersal of iris pigment into the anterior chamber. **D-F**. In contrast, D2.*Tyrp1*^*B6*^*Gpnmb*^*B6 *^mice show no iris pigment disease, transillumination defects or alteration of the anterior chamber.

**Figure 3:**
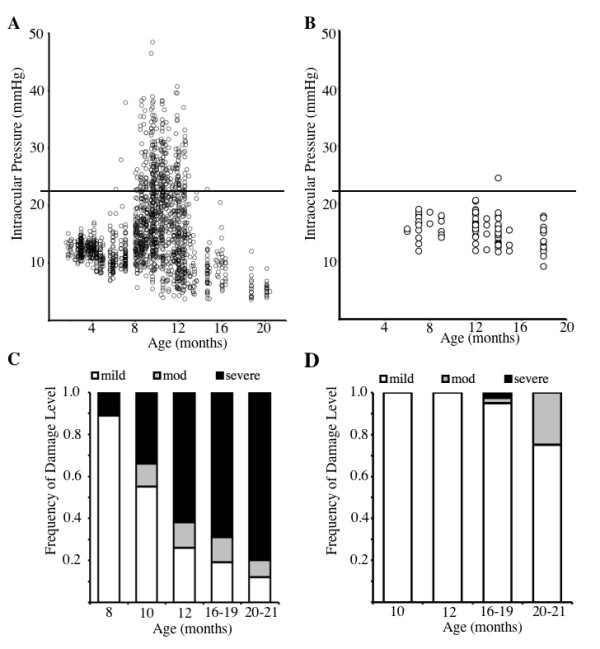
Assessment of IOP levels and glaucoma in D2.*Tyrp1*^*B6*^*Gpnmb*^*B6 *^mice. **A**. IOP profile of a population of D2 mice (1437 IOPs) between 4 and 20 months of age. **B**. IOP profile of 88 D2.*Tyrp1*^*B6*^*Gpnmb*^*B6 *^mice between 6 and 18 months of age. Individual IOP measurements are plotted as circles. The horizontal line indicates 21 mmHg, a value considered to be glaucoma-suspect in people. IOP elevation above 21 mmHg is first observed in D2 mice at around 6 months of age, and peaks at 9–10 months. D2.*Tyrp1*^*B6*^*Gpnmb*^*B6 *^mice show no significant IOP elevation above 21 mmHg. **C**. Previously reported frequencies of optic nerve damage in D2 mice are presented showing clear glaucomatous damage. Approximately 70% of D2 eyes have moderate or severe damage by 12 months of age, and 80% by 16–19 months of age. **D**. In contrast no glaucomatous damage was seen in 18 D2.*Tyrp1*^*B6*^*Gpnmb*^*B6 *^eyes at 12 months of age, and only 5% (3 of 39) of eyes from 16–19 months of age had obvious disease. A total of 72 eyes were analyzed for all ages. Mild nerves have fewer than 5% damaged axons, moderate (mod) nerves have between 5% and 50% axon damage, and severe nerves have greater than 50% axons damaged. Only moderate and severe nerves are considered to show glaucomatous damage, as mild damage is frequently observed in aging non-glaucomatous strains by 10 to 12 months of age. At 12 months of age, moderate damage is very unusual in non-glaucomatous strains and so we consider this degree of damage a sign of glaucoma. Although moderate damage is not common in non-glaucomatous mice, it does occur in more mice of non-glaucomatous strains by 16 to 18 months. Thus, at these older ages, moderate damage may be caused by an unusual degree of age-related RGC demise or by glaucoma. The single case of severe damage is extremely rare in control mice and may or may not represent glaucoma.

As previously reported, the irides of D2 mice with both the *Gpnmb*^*R150X *^and *Tyrp1*^*b *^mutations develop severe pigment dispersion and iris atrophy (Figure 2a–c). However, as expected, the irides of D2.*Tyrp1*^*B6*^*Gpnmb*^*B6 *^mice appear totally normal at all ages. IOP elevation is a key risk factor for glaucoma and occurs in D2 mice as a result of the iris disease. Typically, a population of D2 mice shows elevated IOP levels that peak at around 9–10 months of age (Figure 3a). In contrast, IOPs of D2.*Tyrp1*^*B6*^*Gpnmb*^*B6 *^mice are not elevated compared to young mice at any assessed age (Figure 3b).

D2.*Tyrp1*^*B6*^*Gpnmb*^*B6 *^mice were further assessed for optic nerve damage. The amount of RGC axon damage was determined using a three point damage rating system based on the amount of axon damage detected in the retrorbital portion of the optic nerve [10]. This system has been validated against axon counting [18,19]. 70% of D2 mice have glaucomatous optic nerve damage by 12 months of age (Figure 3c). In contrast, D2.*Tyrp1*^*B6*^*Gpnmb*^*B6 *^show no evidence of glaucomatous damage at 12 months of age (Figure 3d).

D2.*Tyrp1*^*B6*^*Gpnmb*^*B6 *^mice have B6-derived chromosomal regions (congenic intervals) flanking *Tyrp1 *and *Gpnmb *respectively. Together, these B6-derived regions represent up to 3.3% of the genome (Figure 1). To produce an even more closely matched control strain, we developed D2-*Gpnmb*^+^, a second control strain that has no B6-derived congenic intervals. To generate the D2-*Gpnmb*^+ ^strain, the *Gpnmb*^*R150X *^allele of modern day D2 mice was replaced with the *Gpnmb*^+ ^allele from the D2-*sdy *strain (the original wild-type D2 *Gpnmb *allele, see Methods).

### D2-Gpnmb^+ ^mice develop a mild iris disease

**Figure 4:**
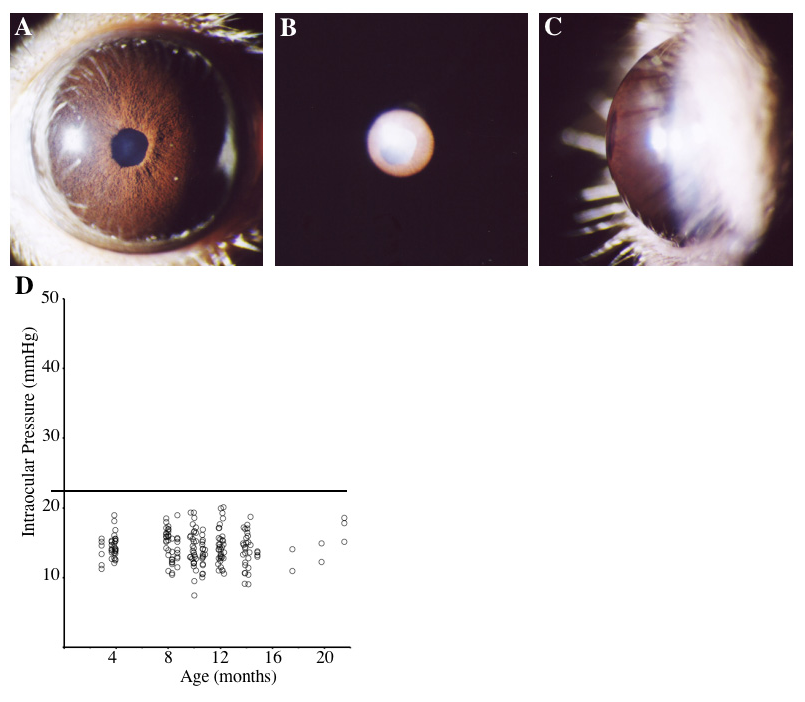
**A-C**. Iris disease and IOP in D2-*Gpnmb*^+ ^mice. **A**. Broad-beam illumination. **B**. Transillumination. **C**. Side view dimensions of the anterior chamber. D2-*Gpnmb*^+ ^eyes show no IPD but have a distinct form of ISA where the iris appears 'roughened'. D2-*Gpnmb*^+ ^eyes have no transillumination defects and normal anterior chamber morphology (more than 20 eyes were analyzed from a variety of ages, images shown here are from a 13 months old mouse). **D**. IOP measurements in D2-*Gpnmb*^+ ^mice. Individual IOP readings are plotted as circles. The solid horizontal line indicates 21 mmHg, a value considered to be glaucoma-suspect in people. No 'high' IOP readings were seen in 97 eyes measured. Also, there is no significant change in IOP levels comparing 4 months old D2-*Gpnmb*^+ ^mice to the 8, 10, 12 or 14 months old groups (p > 0.37 for all comparisons).

D2-*Gpnmb*^+ ^mice were aged and assessed for ISA and IPD. Although wild-type for *Gpnmb*, D2-*Gpnmb*^+ ^mice are homozygous for the *Tyrp1*^*b *^mutation. Therefore, they are expected to develop the ISA iris disease that is caused by this mutation. A mild ISA disease is observed in these mice (Figure 4a–c). The ISA phenotype in D2-*Gpnmb*^+ ^mice is characterized by a change in iris stromal morphology (appearing "roughened" and lacking complexity). However, the iris shows no evidence of IPD, maintains integrity, and is overall very mildly affected when compared to the severe, combined ISA and IPD phenotype of D2 mice.

### D2-Gpnmb^+ ^mice do not develop elevated IOP or glaucoma

**Figure 5:**
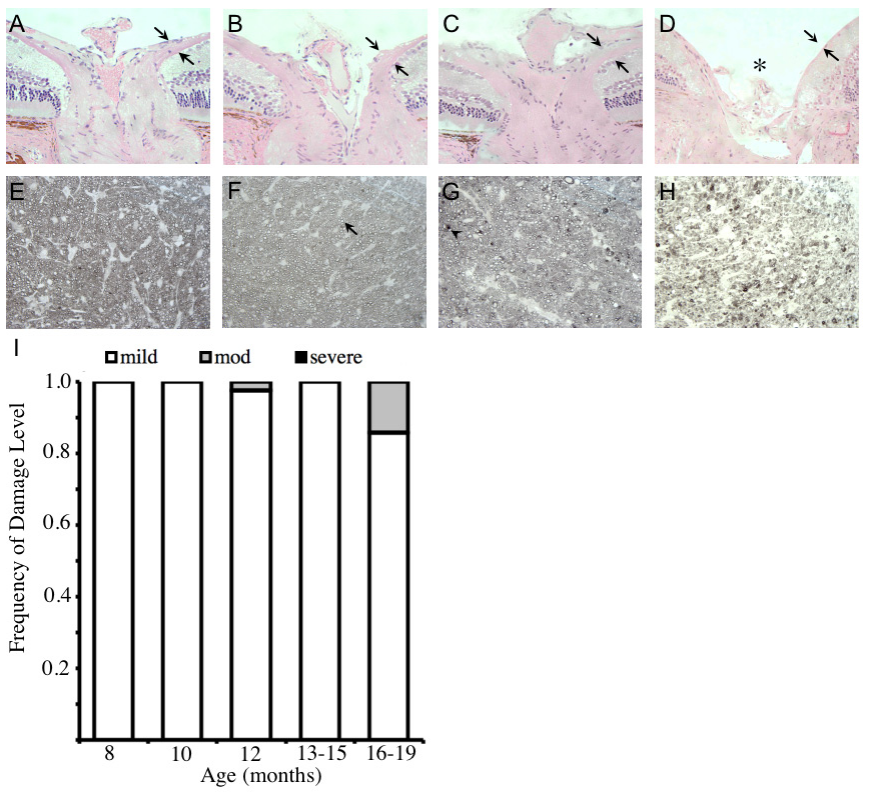
D2-*Gpnmb*^+ ^mice do not develop glaucoma. **A-C**. H&E stained sections of D2-*Gpnmb*^+ ^mice at 4.5 mo (**A**), 12 mo (**B**) and 18 mo (**C**) showing a normal nerve fiber layer (arrows) and no evidence of optic nerve cupping. **D**. H&E section of a 12 months old D2 mouse with severe glaucoma, showing optic nerve cupping (asterisk) and loss of nerve fiber layer (arrows). **E-G**. PPD staining of the retro-orbital optic nerve from a 4.5 mos (**E**), 12 mos (**F**), 18 mos (**G**) D2-*Gpnmb*^+ ^mice. These nerves are healthy with only rare degenerating axons, as is typical for nerves of non-glaucomatous mouse strains. **H**. Severe glaucoma in a 12 mos D2 eye. Healthy axons have clear axoplasm, surrounded by darkly stained myelin (arrow). The sick or dying axons stain grey/black (arrowhead). (**I**) Frequency of glaucomatous optic nerve damage in D2-*Gpnmb*^+ ^mice, using the 3 point grading system (see Methods). No D2-*Gpnmb*^+ ^mice show severe glaucoma, and only 3 eyes show moderate damage. Importantly, these moderately affected nerves only just enter this grade (all had less than 10% axon loss), and were not as severely degenerated as the typical D2 moderate nerve.

IOP was assessed at a variety of ages between 4 months and 23 months of age. The selected ages are relevant to the window of IOP changes observed in DBA/2J mice. There was no age-related increase of IOP in these mice. Additionally, no D2-*Gpnmb*^+ ^mice had IOP values above 21 mmHg, levels considered to be glaucoma-suspect in people (Figure 4d). Consistent with this, we detected no signs of glaucomatous RGC death and optic nerve degeneration in D2-*Gpnmb*^+ ^mice. D2 mice show RGC death, loss of the nerve fiber layer and severe optic nerve cupping by 12 months of age [10]. Retina and optic nerve morphology was examined in 4 eyes taken from D2-*Gpnmb*^+ ^mice at each of 4.5 months, 12 months and 18 months of age (Figure 5a–c). The RGC layer and nerve fiber layer was normal, and there was no evidence of optic nerve head cupping (Figure 5d). Optic nerve damage was further assessed by sectioning a portion of the retro-orbital optic nerve and staining with PPD, a stain that sensitively detects degenerating axons. There was no evidence of glaucomatous optic nerve damage in D2-*Gpnmb*^+ ^eyes taken from a large number of mice between 4.5 and 15 months of age (Figure 5e–i). Importantly, there was no correlation between optic nerve damage and measured IOP level in individual mice and thus the range of pressure seen in D2-*Gpnmb*^+ ^did not affect nerve damage. In addition, we compared axon number in young pre-glaucomatous D2 eyes with aged D2-*Gpnmb*^+ ^eyes, using a previously described counting system [18,19], and there was no significant difference in axon number (51,748 ± 907 for D2, and 52,074 ± 427 for D2-*Gpnmb*^+^, n = 10 for each group, p = 0.7). A moderate form of optic nerve damage was present in one eye from a 12 months old mouse, and 2 eyes from mice over 16 months of age. This may be a very rare case of glaucoma due to the mutant *Tyrp1 *gene, or more likely age-related damage that is not related to glaucoma as a similar degree of damage is observed in other non-glaucomatous mouse strains of a similar age [12,20].

## Discussion

DBA/2J is an established mouse model for inherited glaucoma that shows iris pigment dispersion, IOP elevation and optic nerve degeneration. D2 mice are homozygous for both the *Gpnmb*^*R150X *^and *Tyrp1*^b ^mutations. Both of these mutations are necessary for the severe iris pigment disease that leads to IOP elevation and glaucoma [16]. Although DBA/2J is an experimentally tractable model of glaucoma, it is an inbred mouse strain and so no strain matched DBA/2J controls without glaucoma have been available. Here we describe the generation of two derivative strains of DBA/2J (D2.*Tyrp1*^*B6*^*Gpnmb*^*B6 *^and D2-*Gpnmb*^+^), where either the mutant alleles of both *Gpnmb *and *Tyrp1 *or only *Gpnmb *have been replaced by wild-type versions. D2.*Tyrp1*^*B6*^*Gpnmb*^*B6 *^mice contain a maximum of 3.3% of B6-derived sequence on a D2 background. In the case of D2-*Gpnmb*^+^, the only known difference compared to D2 is a single base change in the *Gpnmb *gene. Neither D2.*Tyrp1*^*B6*^*Gpnmb*^*B6 *^nor D2-*Gpnmb*^+ ^strains develop IOP elevation or glaucoma.

Since determining that the *Gpnmb *mutation recently arose on the DBA/2J background [10], we have produced a further control strain, D2.*Tyrp1*^*B6*^*Gpnmb*^+^, that is currently being characterized. This strain contains a congenic interval flanking *Tyrp1 *but is improved over D2.*Tyrp1*^*B6*^*Gpnmb*^*B6 *^in that it lacks a congenic interval around *Gpnmb *(as the *Gpnmb*^+ ^allele is the ancestral DBA/2J allele). This strain is wild-type for both glaucoma inducing genes and is not expected to develop any D2 glaucoma-associated phenotypes. Although characterization of large numbers of these mice is a lengthy undertaking, initial analyses agree with the findings reported here for D2.*Tyrp1*^*B6*^*Gpnmb*^*B6 *^mice, with no evidence of iris disease, IOP elevation or glaucoma.

Regarding the control strains, the following points are pertinent. Although they lack all iris disease and glaucoma, the D2.*Tyrp1*^*B6*^*Gpnmb*^*B6 *^strain does not typically reproduce as well as the D2-*Gpnmb*^+ ^strain and appears to have an increased incidence of early deaths (based on retrospective observations, but no specific spontaneous death study has been conducted). Although not well characterized, the decreased fecundity appears to be related to the *Tyrp1*^B6 ^allele on a D2 background, since other D2 strains that we have been producing with this allele have exhibited a similar phenotype. These issues do not prevent the use of this strain. However, since the D2-*Gpnmb*^+ ^strain lacks these issues and has no B6 derived interval, we recommend it as a control when studying glaucomatous neurodegeneration. Although the D2-*Gpnmb*^+ ^strain still develops a mild iris disease, glaucomatous nerve damage was clearly absent in the vast majority, if not all, of these mice. Thus, despite the spread in IOP, the highest IOPs were not significant enough to induce glaucomatous axon loss. When using these control strains, it is important to remember that they may still have some undefined but glaucoma relevant phenotype(s).

It is clear that glaucoma is a multifactorial disease and D2 mice are likely to have susceptibility factors in addition to those associated with the *Gpnmb *and *Tyrp1 *genes. Thus, although these strains do not develop glaucoma, as defined by the absence of nerve damage, they may still have other phenotypes that render the D2 background susceptible to glaucoma. Such phenotypes may or may not have contributed to nerve the damage detected in a small percentage of mice.

*Tyrp1 *and *Gpnmb *function in melanosomes, organelles that produce pigment. Manifestation of both IPD and ISA in D2 eyes is dependent upon active pigment production [16,20]. The mechanisms involved in IPD and ISA and the subsequent IOP elevation are complex and in addition to pigment production appear to involve immunity [21,22]. Ongoing functional mouse genetics experiments complemented with a variety of immunological, molecular and cellular techniques are vital to unravel these mechanisms. The control strains described here will be a valuable resource in elucidating the biological mechanisms involved in pigment-related iris disease. In addition to ocular-related diseases, these strains provide an important resource to test the roles of *Tyrp1 *and *Gpnmb *in other diseases. *Tyrp1 *is the most common melanoma antigen and *Gpnmb *is used as a marker for metastatic melanomas [23] and has recently been identified as a potential molecular therapeutic target in patients with glioblastoma multiforme [24]. *Gpnmb *may also be important in antigen presenting cells that control immune responses [21].

To improve our understanding of glaucoma, it is essential that genes and pathways involved in IOP elevation and glaucomatous RGC and optic nerve degeneration are identified. By analyzing gene expression profiles in D2 mice at various ages [25], and conducting subsequent functional tests, it is possible to identify differentially expressed genes important for the onset and/or progression of glaucoma. Individual mouse strains, such as D2, have a unique collection of alleles affecting many biological processes and that affect gene expression levels and protein activity [26]. Therefore, many gene expression differences are observed between D2 and other naturally occurring strains that do not get glaucoma [27]. This can complicate experiments and make it difficult to prioritize genes for subsequent functional testing. Additionally, it is possible that strain differences can mask some expression changes that occur in D2 mice and are relevant to the glaucoma. By enabling analysis of age- and strain-matched control mice, the control strains reported here alleviate the difficulties of controlling experiments using D2 mice.

## Conclusion

The strains describes here will enable the systematic evaluation of the processes involved in iris disease, IOP elevation and glaucoma, work that is likely to provide novel understanding and lead to targets for improved therapeutics for human glaucomas.

## Methods

### Animal husbandry and strain development

Mice were housed in a 14 h light to 10 h dark cycle under previously described conditions [9,15]. The Jackson Laboratory's (Bar Harbor, Maine, United States) pathogen surveillance program regularly screened for pathogens. All experiments were conducted in accordance with the Association for Research in Vision and Ophthalmology's statement on the use of animals in ophthalmic research and were approved by our institutional animal care and use committee. Modern DBA/2J (D2) mice (#000671, see [28]) have mutations in both *Tyrp1 *and *Gpnmb*. Although the majority of included data for modern D2 mice has been previously published, over 300 modern D2 mice were aged and analyzed over the same period of time as the mice of the other strains presented here. For the generation of the D2.*Tyrp1*^*B6*^*Gpnmb*^*B6 *^strain, D2 mice were crossed to C57BL/6J (B6) to create F1s. Progeny carrying the B6 allele of both *Gpnmb *(*Gpnmb*^*B6*^) and *Tyrp1 *(*Tyrp1*^*B6*^) were then backcrossed to D2 for ten generations. Brother/sister matings were then established to generate mice homozygous for *Gpnmb*^*B6 *^and *Tyrp1*^*B6 *^and to maintain a stable D2.*Tyrp1*^*B6*^*Gpnmb*^*B6 *^doubly homozygous colony.

The *Gpnmb*^*R150X *^mutation arose in the early 1980s and became fixed in the ancestors of modern day D2 mice [16]. The *sdy *mutation alters coat color and occurred in DBA/2J (D2) mice in 1983. At that point, the DBA/2J-*Dtnbp1*^*sdy *^strain (hereafter referred to as D2-*sdy*) was separated from the main D2 colony. Genotyping a D2-*sdy *colony for *Gpnmb *in the early 2000s revealed that these mice had an original wild-type allele of *Gpnmb *(*Gpnmb*^+^). To develop the D2-*Gpnmb*^+ ^strain, D2-*sdy *were crossed to modern D2 mice for three generations. We are continuing to backcross D2-*Gpnmb*^+ ^to modern day D2 to further reduce the possibility of D2-*Gpnmb*^+ ^mice harboring unknown genetic differences compared to modern day D2 mice. These higher generation D2-*Gpnmb*^+ ^mice will be provided to the community. (They are being accepted for distribution by Jackson Laboratory mouse resources and will become strain DBA/2J-*Gpnmb*^+^/SjJ with stock # 007048). However, to hasten characterization of mice with a D2 genetic background and a wild type allele of *Gpnmb*, brother/sister matings that did not carry the *sdy *mutation were selected to establish the D2-*Gpnmb*^+ ^strain that is homozygous for the wild-type *Gpnmb *allele and characterized here. These matings also produced the mice for clinical examinations, IOP measurements and assessment of glaucomatous damage. Analysis of 102 microsatellite markers (average spacing 13.25 cM) revealed no allelic differences between D2-*Gpnmb*^+ ^and modern day D2.

### Genotyping

The *Gpnmb*^*R150X *^mutation creates a novel *Pvu*II enzyme site [16]. DNA isolated from tail snips was used to PCR amplify the 125 bp region surrounding the mutation using primers nmb7 (CTACAACTGGACTGCAGGGG) and nmb8 (AGCTCCATTTCTTCCATCCA). The resulting product was digested with *Pvu*II (NEB). The presence of the mutation is indicated by the presence of two bands of 50 bp and 75 bp. Identification of B6-derived sequence that included *Tyrp1 *on Chromosome 4 was achieved using the flanking polymorphic markers *D4Mit178 *(D2 = 170 bp, B6 = 146 bp) and *D4Mit327 *(D2 = 92 bp, B6 = 106 bp). Identification of B6-derived sequence that included *Gpnmb *on Chromosome 6 was achieved using the flanking polymorphic markers *D6Mit74 *(D2 = 150 bp, B6 = 160 bp) and *D6Mit355 *(D2 = 106 bp, B6 = 124 bp). The extent of the B6-derived region was analyzed using polymorphic markers spanning Chromosomes 4 and 6 (see [29]).

### Clinical examinations

We examined eyes at 2–24 months of age with a slit-lamp biomicroscope (Haag-Streit) and photographed them with a 40× objective lens. Phenotypic assessment of iris stromal atrophy, dispersed pigment and transillumination was carried out as described previously [9,15,20].

### IOP and optic nerve assessment

Mice were acclimatized to the procedure room, anesthetized by using an intraperitoneal injection of ketamine/xylazine mixture and IOP measured as previously described [30]. Optic nerve cross sections were examined for glaucomatous damage using a modified paraphenylenediamine (PPD) staining protocol to stain the myelin sheath of all axons and the axoplasma of damaged axons as described previously [18]. Optic nerves were assessed for glaucomatous damage using a non-biased three point grading system that has been validated against axon counting [18,19]. Axon counts were carried out as previously described [18,19]. 10 randomly selected D2 eyes (pre-glaucomatous, 3 to 4 months) and 10 randomly selected D2-*Gpnmb+ *eyes (no glaucoma, 10.5 to 11 mos) were counted.

### Histology

Eyes were fixed in 4% paraformaldehyde (PFA) overnight at 4°C and stored in 0.4% PFA at 4°C. For general morphology, eyes were processed, embedded in plastic, sectioned and stained with Hemotoxylin and Eosin.

## Authors' contributions

GH carried out optic nerve assessment of strains, coordinated data collection and analysis and prepared the manuscript. RL participated in the design of the experiment, carried out optic nerve assessment of strains and helped write the manuscript. JKM performed the axon counts. LW organized the generation of the strains, IOP measurements and harvesting. IC carried out the IOP measurements. RS carried out optic nerve assessment. MA participated in the design of the experiment and strain generation, carried out clinical assessments of the mice, and helped write the manuscript. SJ conceived the study and participated in its design and coordination and oversaw all components including manuscript preparation. All authors read and approved the final manuscript.
